# Profile of a Food-Insecure College Student at a Major Southeastern University: A Randomized Cross-Sectional Study

**DOI:** 10.3390/nu15051108

**Published:** 2023-02-23

**Authors:** Cedric Harville, Delores C. S. James, Arné Burns

**Affiliations:** 1 Department of Applied Health, Southern Illinois University-Edwardsville, Campus Box 1126, Edwardsville, IL 62026, USA; 2 Health Education & Behavior, University of Florida, 1864 Stadium Road, Gainesville, FL 32603, USA

**Keywords:** food insecurity, college students, SNAP, WIC, government housing, free/reduced lunch, food shortage contacts

## Abstract

Ten percent of Americans are food-insecure. Few known studies have accessed college food insecurity via random sampling. An online cross-sectional survey (*n* = 1087) was distributed via email to a random sample of undergraduate college students. Food insecurity was determined by the USDA Food Security Short Form. Data were analyzed using JMP Pro. Results: Thirty-six percent of the students were food-insecure. Most food-insecure students were enrolled full-time (93.6%), female (81.2%), received financial aid (77.9%), lived off-campus (75.0%), non-white (59.6%), and employed (51.7%). Food-insecure students had a significantly lower GPA (*p* < 0.001 *), were more likely to be non-white (*p* < 0.0001 *), and were more likely to have received financial aid compared to food-secure students (*p* < 0.0001 *). Food-insecure students were significantly more likely to have lived in government housing, had free or reduced lunch, used SNAP and WIC benefits, and received food from a food bank during childhood (*p* < 0.0001 * for all). Food-insecure students were significantly less likely to report that they experienced a food shortage to counseling and wellness personnel, a resident assistant, and their parents (*p* < 0.05 * for all). Discussion: College students might be at greater risk for food insecurity if they are non-white, first-generation students, employed, on financial aid, and have a history of accessing government assistance during childhood.

## 1. Introduction

Food insecurity is a global health concern. A total of 13.5 million Americans (10.2%) have experienced food insecurity at some point within the last year [1]. Food insecurity is defined by the United States Department of Agriculture (USDA) as the “access by all people at all times to have enough food for an active, healthy life” [1]. Trends in US food insecurity show that the current rate is at the lowest point since 1999 [1].

The USDA does not directly measure food insecurity among college student populations, but rather US adults, households, and children [2]. However, quantifying college student food insecurity in the published literature has been carried out via validated USDA scales such as the six- and ten-item food security forms [2]. College student food insecurity in the published literature prior to the Coronavirus 2019 (COVID-19) pandemic has shown rates as low as 14%, and nearly 59% at its highest [3,4,5,6,7,8,9,10,11,12,13,14,15,16,17,18,19,20,21,22,23]. Interestingly, most studies related to college food insecurity have used non-probability sampling methods [3,4,5,6,7,8,9,10,11,12,13,14,16,17,18,19,20,21,23]. Few studies [15,22] obtained a random sample of college students to assess the prevalence of food insecurity, which is generally viewed as the “gold standard” for sampling [24].

Qualitative studies have sought to explore the specific factors that might lead to college student food insecurity and found that food-insecure college students are having to sacrifice their food budgets due to having fixed bills [25]. Additionally, incidental expenses such as car troubles and medical bills occur, which results in them skipping/stretching meals and purchasing cheaper and less healthy foods [25]. One study that explored the psychosocial effects of food insecurity found that students were frustrated with their institution due to a lack of resources available for struggling students and having a feeling of hopelessness due to an inability to be employed due to potential excessive work hours [26].

This late adolescence and early adulthood period is characterized by those of college age and possibly living on their own for the first time [27]. Consequently, college students start to become independent (financially) as they are forced to develop and shape their own lifestyle, eating, and health behaviors [27]. Studies have associated college student-related food insecurity risk factors due to limited income, the increased cost of college and expenses, increased credit card reliance and debt, and limited access to supplemental programs such as the Supplemental Nutrition Assistance Program (SNAP) [3]. However, no known studies have linked students’ possible family history of reliance on government assistance as a possible indicator of collegiate food insecurity.

Understanding the profile of a food-insecure college student can potentially provide better insight to colleges and universities of how to pinpoint or target possible students that might be food-insecure. The goal of this study was to describe the profile of a food-insecure college student by assessing the prevalence of food insecurity, the history of government assistance, and food shortage contacts by food security status among a random sample of enrolled college students at a major southeastern university.

## 2. Materials and Methods

### 2.1. Sample

A self-administered online survey via Qualtrics was sent to a simple random sample of 5000 currently enrolled University of Florida (UF) undergraduate students. The total undergraduate population at UF was 35,043 (female: 19,355 (55.2%), male: 15,688 (44.8%)). A request was made to the UF Office of Institutional Planning and Research for the simple random sample of currently enrolled undergraduate students. The Excel file received only contained student emails with no other identifying information provided. No incentives were provided to students for participation. Data were collected from 25 April to 3 June 2017. In order to participate in the study, students had to: (1) be currently enrolled (full-time or part-time) at the University of Florida, (2) be undergraduate students, (3) have the ability to read and write in English, and (4) have a valid UF email address. Overall, 1087 students completed the survey, resulting in a response rate of 21.7%.

Potential respondents were emailed an introductory letter describing the study, potential benefits and harms of participation, Institutional Review Board (IRB) contact information and approval/number, and an individualized/personalized link to the survey. Additionally, respondents were not offered an incentive to take the survey. Respondents had two weeks to complete the survey after they started. Consistent with the Dillman Total Design Method of survey administration, after the introductory email, three automated personalized reminder emails were sent to non-respondents every three days [28]. Similarly, automated emails were sent to respondents who started but did not complete the survey. Respondents who completed the survey received an automated “thank you” from Qualtrics. Respondents that had missing data from the food security items were excluded from the analysis. This study was approved by the UF IRB.

### 2.2. Measure of Food Insecurity

The six-item food security scale is an instrument created by the USDA developed to determine the level of food security of individuals [29]. The six-item Food Security Short Form has been validated in previous food insecurity studies with college students [4,5]. Individuals were scored based on the number of “affirmative” responses provided. Affirmative responses consisted of the reported responses “sometimes”, “often”, and “yes”. Individuals with affirmative responses that led to raw scores of 2 or greater were considered “food-insecure”. For any score that was less than 2, participants were considered “food-secure”. This scoring was consistent with previous literature that categorized food security status into two different groups “food-insecure” and “food-secure” [4,5].

There are six-items from the USDA Food Security Short Form which measure food insecurity experienced within the last 12 months. These six items are:

- “In the last 12 months, the food that (I/we) bought just didn’t last, and (I/we) didn’t have money to get more.”
- “In the last 12 months, (I/we) couldn’t afford to eat balanced meals.”
- “In the last 12 months, since last (name of current month), did (you/you or other adults in your household) ever cut the size of your meals or skip meals because there wasn’t enough money for food?”
- “How often did this (cut the size of or skip meals) happen—almost every month, some months but not every month, or in only 1 or 2 months?”
- “In the last 12 months, did you ever eat less than you felt you should because there wasn’t enough money for food?”
- “In the last 12 months, were you ever hungry but didn’t eat because there wasn’t enough money for food?”

### 2.3. Measure of Government Assistance

Respondents were asked “if their family ever obtained government assistance during childhood (government housing, free/reduced lunch in school, SNAP, Women, Infants, and Children (WIC), and food banks)”. Respondents had the option to “choose all that apply” from the listed options.

### 2.4. Measures of Food Shortage Contacts

For the last item, respondents were asked to “choose all that apply” as to whether they felt comfortable discussing with a parent, roommate, counseling and wellness personnel, advisor/mentor, resident assistant, or professor if they were running short on food.

### 2.5. Data Analysis

The data for this study were analyzed using JMP Pro 12 (SAS Institute Inc., Cary, NC, USA, 1989–2021) software. Descriptive statistical analyses were used to provide group percentages and compare differences among the individual variables. Sociodemographic characteristics that were measured include: current college classification (lower class (freshman/sophomore), upper class (junior/senior)), gender identity (male/female), race (white/non-white), current age, parental status, first-generation college student, current grade point average (GPA), enrollment status (full-time/part-time), relationship status (married/single), work status (employed/unemployed), housing status (on-campus/off-campus), US citizenship (US citizen/non-US citizen), years in the US, distance/online student, fraternity/sorority member, on financial aid, estimated student debt owed, knowledge of campus food bank, and ever accessed the campus food bank. Sociodemographic characteristics were compared based on food security status using chi-square (*X*^2^) with odds ratios (OR) and 95% confidence intervals (CI) and independent samples t-tests were used for continuous variables to compare food-insecure and food-secure students.

Categorization of participants based on the individual total scores on the USDA Food Security Short Form determined their food security status (0–1 = “food-secure”, ≥2 = “food-insecure”). Food security status differences were calculated using *X*^2^ for all. All statistical tests had significance set at *p* ≤ 0.05.

## 3. Results

### 3.1. Sample Characteristics

The mean age for college students was 20.77 ± 3.43. Most (78.1%, *n* = 838) were female. There was a nearly even split of white (50.1%, *n* = 540) and non-white (49.9%, *n* = 537) students in this study. Most of the students were upper class (59.1%, *n* = 632). Majority of students were single (97.4%, *n* = 1046), did not have children (97.9%, *n* = 1050), and were born in the US (86%, *n* = 924). Students that were not born in the US (14%, *n* = 150) had lived in the US for on average 12.85 ± 5.33 years. Most students were enrolled full-time (93.5%, *n* = 999) and not in a distance education program (94.9%, *n* = 1012), were not members of a fraternity/sorority (80.9%, *n* = 870), were not first-generation college students (67.3%, *n* = 721), and lived off-campus (60.2%, *n* = 647).

#### 3.1.1. Food Insecurity

Students responded to a total of six items to measure their individual level of food insecurity. Out of 1087 total responses to all items, 36.1% (*n* = 392) were found to be food-insecure within the past 12 months. Individual student responses to the USDA Food Security Short Form can be found in Table 1.

Food insecurity differences were found based on race. Most of the students that were food-insecure were non-white (59.6%, *n* = 233), compared to the white students (40.4%, *n* = 158). Compared to food-secure students, food-insecure students were found to be significantly more likely to be non-white students (*χ*^2^(1) = 23.25; OR = 0.54; 95% (CI) = 0.42–0.69; *p* < 0.0001 *).

#### 3.1.2. Food Insecurity and Food Bank Usage

Over half of all college students (56.5%, *n* = 607) were aware a campus food bank existed. For the food-insecure students, 61.6% (*n* = 241) were aware that a food bank existed on their college campus. Compared to food-secure college students, those that were food-insecure were found to be significantly more likely to be aware that a campus food bank existed (*χ*^2^(1) = 6.69; OR = 0.71; 95% (CI) = 0.56–0.92; *p* < 0.01 *). However, only 9.1% of all students had ever accessed the campus food bank. In comparison, 13.3% (*n* = 32) of food-insecure students had ever accessed the campus food bank. Compared to food-secure college students, those that were food-insecure were found to be significantly more likely to access the campus food bank (*χ*^2^(1) = 8.63; OR = 0.44; 95% (CI) = 0.25–0.77; *p* < 0.01 *).

#### 3.1.3. Campus Meal Plan

Most students did not have a campus meal plan (52.3%, *n* = 563). Only 33.8% (*n* = 173) of food-insecure students had a campus meal plan, compared to 66.2% (*n* = 339) of food-secure students. No significant differences in access to a campus meal plan by food security status were found (*p* > 0.05).

#### 3.1.4. GPA

The average GPA was 3.45 ± 0.39 for all participants. The mean GPA of all food-insecure students was 3.36 ± 0.42. Significant differences in GPA were found based on food security status (t(1039) = 34.78; *p* < 0.001). Food-insecure college students had a significantly lower GPA compared to food-secure students (3.36 ± 0.42 vs. 3.50 ± 0.36).

#### 3.1.5. Student Employment and Volunteer Activities

Most students (53.7%, *n* = 575) were not employed. Students that reported working averaged 18.49 ± 10.07 h per week. A majority of food-insecure students were employed (51.7%, *n* = 201) during college. However, a majority of food-secure students were not employed (56.8%, *n* = 387). Food-insecure college students were significantly more likely to be employed while in school (*χ*^2^(1) = 7.19; OR = 0.71; 95% (CI) = 0.55–0.91; *p* < 0.01 *) compared to food-secure students. Food-insecure college students currently worked an average of 18.77 ± 9.78 h per week compared to food-secure students, working 18.30 ± 10.29 h per week. No significant differences in college student hours of employment per week were found based on food security status (*p* > 0.05).

College students reported that they participated in volunteer activities for on average 8.64 ± 6.63 h per week. Food-insecure students were significantly more likely to participate in volunteer activities compared to food-secure students (9.42 ± 6.96 vs. 7.58 ± 6.03; t(719) = 13.73; *p* < 0.001).

#### 3.1.6. Student Financial Aid and Debt

Most students were currently receiving financial aid (65.1%, *n* = 698), with an average loan debt of USD 13,589.48 ± 13,209.02. A majority of food-insecure and food-secure students were currently receiving financial aid (77.9% vs. 57.6%). Food-insecure students were significantly more likely to receive financial aid compared to food-secure students (*χ*^2^(1) = 44.26; OR = 0.39; 95% (CI) = 0.29–0.52; *p* < 0.0001 *). Food-insecure students had a higher loan debt compared to food-secure students, however no significant differences were found (USD 13,999.04 ± 13,242.38 vs. USD 13,194.40 ± 13,198.23).

#### 3.1.7. Marital Status

Most students were single (97.4%, *n* = 1046). Majority of food-insecure students (98.5%, *n* = 384) and food-secure students were also single (96.8%, *n* = 662). There were no significant differences based on marital status (*p* > 0.05).

#### 3.1.8. US Citizenship

Most students were born in the US (86.0%, *n* = 924). Majority of food-insecure students (84.4%, *n* = 329) and food-secure students were born in the US (87.0%, *n* = 595). Those that were not born in the US had lived in the US for an average of 12.85 ± 5.33 years. Food-insecure students had lived in the US for fewer years compared to food-secure students not born in the US (12.62 ± 5.07 vs. 13.02 ± 5.54 years). There were no significant differences in mean years among food-insecure and food-secure students living in the US (*p* > 0.05).

#### 3.1.9. First-Generation Student

Most (67.3%, *n* = 721) students were not first-generation students. Most of the food-insecure students (61.3%, *n* = 239) and food-secure students (70.7%, *n* = 482) were not first-generation college students. Food-insecure students were significantly more likely compared to food-secure students to be first-generation students (*χ*^2^(1) = 9.94; OR = 1.52; 95% (CI) = 1.17–1.98; *p* < 0.01 *).

#### 3.1.10. Online/Distance Education

Most students were not in an online/distance education program (94.9%, *n* = 1012). Majority of food-insecure students (96.1%, *n* = 371) and food-secure students were not online/distance education students (94.3%, *n* = 641). There were no significant differences among food-insecure and food-secure students in online/distance education programs (*p* > 0.05).

#### 3.1.11. Current Residence

Most students lived off-campus (74.2%, *n* = 798). Majority of food-insecure students (73.4%, *n* = 287) and food-secure students lived off-campus (74.7%, *n* = 511). There were no significant differences among food-insecure and food-secure students based on current residence (*p* > 0.05).

#### 3.1.12. Fraternity/Sorority

Most students were not members of a fraternity/sorority (80.9%, *n* = 870). Majority of food-insecure students (82.4%, *n* = 322) and food-secure students were not members of a fraternity/sorority (80.1%, *n* = 548). There were no significant differences based on membership in a fraternity/sorority among food-insecure and food-secure students (*p* > 0.05).

### 3.2. Food Insecurity and Government Assistance

Participants were asked to “choose all that apply” based on five government assistance programs participated in during childhood. Most students never lived in government housing (94.8%, *n* = 1019), never received food from a food bank (96.0%, *n* = 1043), never received WIC (90.8%, *n* = 987), never accessed food stamp/SNAP benefits (85.0%, *n* = 924), and never received free school lunch/meals (72.4%, *n* = 787). Food-insecure students were significantly more likely to have lived in government housing, had free or reduced lunch, used SNAP WIC benefits, and received food from a food bank during childhood (*p* < 0.0001 * for all). See Table 2 for distributions.

### 3.3. Food Insecurity and Food Shortage Contacts

Participants were asked to “choose all that apply” based on who they were comfortable telling that they were short on food. Most students reported they were comfortable telling a parent (79.8%, *n* = 867) that they were experiencing a food shortage. Other reported responses included: telling a roommate (44.1%, *n* = 479), counseling and wellness personnel (20.2%, *n* = 220), advisor/mentor (9.9%, *n* = 108), resident assistant (5.6%, *n* = 61), and a professor (3.7%, *n* = 40). Food-insecure students were significantly less likely to report that they experienced a food shortage to counseling and wellness personnel, a resident assistant, and their parents (*p* < 0.05 * for all). See Table 3 for all other variables.

## 4. Discussion

### 4.1. Prevalence of Food Insecurity

In the current study, 36.1% of college students were found to be food-insecure. The prevalence of food-insecure college students at this university was nearly four times greater compared to the entire state of Florida (36.1% vs. 9.9%), and three times greater than the food-insecure population in Alachua County, Florida (36.1% vs. 13.4%), where the study was conducted [1,30]. Shockingly, this prevalence was over three times higher than the general US population (36.1% vs. 10.2%) [1].

### 4.2. Profile of a Food-Insecure College Student

The current study found many differences in the demographics of food-insecure students compared to the total sample. Food-insecure college students in this study were significantly more likely to be non-white, on financial aid, employed, with lower GPAs, and first-generation college students compared to those that were food-secure. Consistent with the previous literature of food-insecure college students, those individuals that were non-white were more likely than white students to face food insecurity at a higher prevalence rate [3,5,6,7,8,16,19,22]. Additionally, GPA was found to be significantly lower in food-insecure students compared to those that were food-secure [4,7].

#### 4.2.1. Employment among Food-Insecure College Students

Employment status and hours volunteered per week were found to have significant differences in food security status in the current study. Similarly, one study found that college students were more likely to be food-insecure if they were employed while in school and averaged 18 h of work per week [6]. Interestingly, one study that measured coping strategies among food-insecure college students found that nearly 85% sought employment or worked extra hours in order to pay for food and reduce the burden experienced by being food-insecure [31].

#### 4.2.2. Off-Campus Food-Insecure College Students’ Food Access

Off-campus students represented most of the college students within the study. Off-campus college students represented three times as many of the food-insecure students within the study compared to on-campus students (75% vs. 25%). Off-campus students could possibly experience food insecurity due to a number of factors. First, these students are less likely to have campus meal plans (33.8% vs. 66.2%), which possibly forces them to spend money that might not otherwise be allocated specifically for food, but rather money for books and other college-related expenses [25]. Additionally, students with access to the campus meal plans might not cover enough meals to feel secure towards the middle and end of the semesters. Only two options of meal plans are offered to off-campus students (block and flex only) [32]. Off-campus students have to make plans to cover the lack of meals available to them, whether they pay for food using cash or grocery shop. In comparison, the meal plan options for on-campus residents range between 5-day and 7-day unlimited swipe access [32]. A New York Times article discussed the term, “casual swipes”, where college students who might have extra meals/dollars leftover near or at the end of the semester swipe their cards to assist classmates that might be out of meal plan credits [33]. This might be an option for off-campus students experiencing a food shortage toward the end of the semester. Some on-campus students might access the meal plan option and use their friend’s card who live on-campus in the dining hall to receive a meal. Additionally, if the on-campus students have to spend their excess flex dollars that will not roll over to the next academic year, off-campus students could take advantage of obtaining a meal(s) with a friend.

#### 4.2.3. Food-Insecure Students’ Food Shortage Contacts

Being a college student often comes with the notion of the “silent struggle”, representing the stereotypical college student that might be short on food or barely surviving on limited options [34]. In the current study, most students reported that they would tell their parents if they were short on food. Food-insecure students were significantly less likely to tell their parents that they were short on food. Interestingly, students were found to be less likely to tell their parents about a food shortage because of the perception by parents that it was normal [34]. Second, food-insecure students expressed that the shame in telling their parents would be attached because they would be admitting to not being able to provide for themselves [34]. Third, food-insecure students believed they were adults, and that their parents have their own lives and life situations to deal with [34]. These feelings might be shared by food-insecure students in the current study, especially those that might be first-generation students and those that might have grown up on government assistance and had to access a food bank on occasion(s). Additionally, along the same lines, in the current study, food-insecure college students were significantly more likely to have knowledge of and access the campus food bank. Lastly, food-insecure students were significantly more likely to be on financial aid and employed. These factors might have an impact on the “silent struggle” food-insecure college students deal with on a daily basis with regards to managing dealing with food insecurity. Having to take on and manage more personal debt, maintaining employment, and searching for donated food at food banks all appear to be characteristics of food-insecure students in this representative sample. In the current study, college students reported that they would tell their college roommate if experiencing a food shortage, which was second to parents. Although there was no statistically significant difference between food-insecure and food-secure students, college roommates represent a possible resource to alleviate food insecurity. Henry [34] found that sharing food between roommates was a coping strategy for food insecurity. However, conflicting results in quantitative studies show that college students living with roommates were significantly more likely to be food-insecure compared to living with parents/relatives [7]. Living off-campus allows students to live at a reduced cost. Dividing the cost of rent and utilities with a roommate could possibly help students have more affordable living, but at the same time that cost might be offset by the need to pay for transportation to and from school, etc. Additionally, students that have roommates may not necessarily pool their resources to share food and have communal meals. The main reason for living together is to divide the cost of rent and utilities, whereas students are “on their own” when it comes to food.

One Canadian study of food-insecure college students found that nearly 76% received food from a relative or went to a relative’s home for a meal [31]. Studies suggest that parents and relatives might have a positive impact on helping college students be more food-secure, especially among lower class students [14]. Similarly, the current study found parents to be a good choice for contacts for lower class students. This might suggest that although college is the first step towards independence for students, as a group, freshman and sophomore students might be more reliant on parents and family members for resources that assist in making it through the first few years in college.

## 5. Conclusions

The current study assessed the prevalence of food insecurity among a random sample of undergraduate college students. Based on the results of this study, we can conclude that a food-insecure student is likely to be a non-white, first-generation college student, have a history of being on government assistance, be employed, be on financial aid, have knowledge of and have accessed food bank(s), and have a lower GPA. With over one-third of the sample of students found to be food-insecure, colleges must do a better job addressing possible hunger concerns on their campuses. In society (outside of the academic setting), food banks and pantries are generally used to help mitigate food security concerns among the general population.

College administrators might need to consider this as a possible option for their individual campuses. Most immediately, more work needs to be done to get the word out among students regarding how to access the local food pantry on campus. This can be carried out during orientations, with flyers placed strategically around heavily popular campus hangouts, and among the resident assistants. Additionally, health educators and dietitians might be a resource for college students to access outside of the college campus to access food and qualify for SNAP and other government assistance. Students might be able to qualify for programs such as SNAP and WIC, provided they meet the familial, income, and employment requirements. Food-insecure students might have had a family history of accessing these services, and therefore any stigma that might be associated with governmental assistance could be limited.

On a basic level, the idea that being a hungry college student is normal should be debunked. Additionally, parents of college students need to be more educated about food insecurity on campus and know that students being hungry is not normal. During preview and campus orientation sessions, campus health educators can use this time to reach out to parents and let them know of resources in and around campus that can help students.

Although parents were reported as the most likely to be contacted in case of a food shortage, it is interesting to note that food-insecure students were less likely to report parents as a resource. This finding might have implications for those food-insecure individuals who might not have the ability to tell or ask their parents for assistance in dealing with their food insecurity. Food-insecure students might not ask for assistance from their parents because their parents themselves might not have the means or ability to assist them. It is also possible that these food-insecure students might also have parents that are currently dealing with food insecurity themselves.

The current exploratory study potentially adds to the growing body of literature regarding food insecurity on college campuses. Female students represented 78% of respondents. Despite using simple random sampling methods, future studies should be mindful of possible selection bias. Future food insecurity college studies could advance by being aware of sampling methodology that reduces the chance of bias. All but two prior research studies examining food insecurity on college campuses were cross-sectional. Having longitudinal studies to follow students over a four-year college education could tell a more impactful story as well as better understand what resources student’s access to address their food insecurity. These studies can also provide insight into how potential involvement of campus and governmental resources might mitigate and reduce the food insecurity that student’s experience.

## Figures and Tables

**Table 1:** Student responses to the USDA Food Security Short Form.

Food Security Items	*n*	%
“The food that I bought just didn’t last, and I didn’t have money to get more.”		
Often true	59	5.5
Sometimes true	314	29.2
Never true	666	62.0
Don’t know	36	3.3
In the last 12 months, “I couldn’t afford to eat balanced meals.”		
Often true	99	9.2
Sometimes true	285	26.6
Never true	658	61.3
Don’t know	31	2.9
In the last 12 months, since last (name of current month), did you ever cut the size of your meals or skip meals because there wasn’t enough money for food?		
Yes	278	25.9
No	760	70.8
Don’t know	36	3.3
How often did this happen—almost every month, some months but not every month, or in only 1 or 2 months?		
Almost every month	63	22.7
Some months but not every month	115	41.4
Only 1 or 2 months	86	30.9
Don’t know	14	5.0
In the last 12 months, did you ever eat less than you felt you should because there wasn’t enough money for food?		
Yes	236	22.0
No	802	74.8
Don’t know	34	3.2
In the last 12 months, did you ever eat less than you felt you should because there wasn’t enough money for food?		
Yes	197	18.4
No	843	78.6
Don’t know	33	3.0

**Table 2:** Chi-square analysis of childhood access to government assistance based on food security status of undergraduate college students.

Government Assistance	Yes (%)	No (%)	OR (95% CI)	*X* ^2^	*p*
Free school lunch					
Food-secure	140 (20.1)	555 (79.9)	2.74 (2.08–3.59)	53.60	<0.0001 *
Food-insecure	160 (40.8)	232 (59.2)			
SNAP					
Food-secure	78 (11.2)	617 (88.8)	2.19 (1.56–3.07)	21.52	<0.0001 *
Food-insecure	85 (21.7)	307 (78.3)			
WIC					
Food-secure	37 (5.3)	658 (94.7)	3.41 (2.22–5.22)	34.66	<0.0001 *
Food-insecure	63 (16.1)	329 (83.9)			
Food bank					
Food-secure	13 (1.9)	682 (98.1)	4.51 (2.33–8.72)	23.52	<0.0001 *
Food-insecure	31 (7.9)	361 (92.1)			
Government housing					
Food-secure	5 (0.70)	679 (99.3)	0.20 (0.07–0.56)	11.64	<0.0001 *
Food-insecure	14 (3.6)	377 (96.4)			

* *p* < 0.05.

**Table 3:** Chi-square analysis of contacts for college students if experiencing a food shortage, by food security status.

Food Shortage Contacts	Yes (%)	No (%)	OR (95% CI)	*X* ^2^	*p*
Parents					
Food-secure	579 (83.3)	116 (16.7)	0.55 (0.41–0.75)	15.03	0.0001 *
Food-insecure	288 (73.5)	104 (26.5)			
Roommate					
Food-secure	316 (45.5)	379 (54.5)	2.19 (1.56–3.07)	21.52	<0.0001 *
Food-insecure	163 (41.6)	307 (58.4)			
Counseling & Wellness					
Food-secure	160 (23.0)	535 (77.0)	0.60 (0.44–0.84)	9.24	<0.01 *
Food-insecure	60 (15.3)	329 (84.7)			
Advisor/Mentor					
Food-secure	73 (10.5)	622 (89.5)	0.84 (0.55–1.28)	0.69	0.40
Food-insecure	35 (8.9)	357 (91.1)			
Resident Assistant					
Food-secure	47 (6.8)	648 (93.2)	0.51 (0.28–0.94)	4.82	0.03 *
Food-insecure	18 (3.6)	378 (96.4)			
Professor					
Food-secure	25 (3.6)	670 (96.4)	1.06 (0.56–2.05)	0.04	0.85
Food-insecure	15 (3.8)	377 (96.2)			

* *p* < 0.05.

## Data Availability

The data presented in this study are available upon request from the corresponding author. The data are not publicly available due to FERPA regulations.
